# Regional Water Resources Security Evaluation Based on a Hybrid Fuzzy BWM-TOPSIS Method

**DOI:** 10.3390/ijerph17144987

**Published:** 2020-07-10

**Authors:** Yan Tu, Kai Chen, Huayi Wang, Zongmin Li

**Affiliations:** 1 School of Safety Science and Emergency Management, Wuhan University of Technology, Wuhan 430070, China; ck18855442955@whut.edu.cn (K.C.); huayi@whut.edu.cn (H.W.); 2 School of Business, Sichuan University, Chengdu 610065, China; lizongmin@scu.edu.cn

**Keywords:** regional water resources security, evaluation, BWM, TOPSIS, triangular fuzzy linguistic sets

## Abstract

Nowadays, water resource security is becoming increasingly prominent, and this problem is a primary bottleneck restricting China’s future sustainable development. It is difficult to come to a unified conclusion on water resources security, and applications of highly feasible evaluation methods are lacking in practice. In this paper, a novel evaluation methodology is proposed for regional water resources security evaluation. First, water security is divided into two aspects: water quantity security and water quality security. The disposal rate of harmless household garbage, the excellent water resources proportion, and the functional water body loss proportion are creatively considered as indicators of water quality security in the evaluation system. In addition, a Technique for Order Preference by Similarity to Ideal Solution (TOPSIS) method is used to evaluate the water security levels in different regions. For distinguishing the importance of different indicators, a Best–Worst Method (BWM) is employed to calculate the indicator weights, as triangular fuzzy linguistic sets can more flexibly describe the preferences of decision makers (DMs) regarding the indicators; therefore, it is embedded in BWM to determine indicator weights. Moreover, the fuzzy BWM-TOPSIS method is applied to evaluate the water security levels of six regions in North China, a comparison analysis with the equal weight TOPSIS method as well as the fuzzy BWM-AHP method, and a sensitivity analysis for indicator weights are presented to illustrate the effectiveness of this proposed method. Finally, some suggestions based on the evaluation results are given for effective and rational utilization of water resources in North China.

## 1. Introduction

Since the beginning of the 21st century, water resource security issues have increasingly restricted development all over the world [1,2]. As the most important renewable resource for human survival, water has not been valued by the non-renewable energy sources sector, including oil and natural gas, because of its renewability and large reserves. However, it must be acknowledged that water resources play an irreplaceable role in industrial construction, daily life, agricultural irrigation, and protection from fires in the ecological environment. With the rapid development of China’s economy, water pollution has increasingly intensified, forming a compounded water shortage situation from both quality and quantity perspectives. China’s water problem has become one of the main obstacles to the country’s development and modernization [3].

According to the 2018 World Water Development Report released by the United Nations at the 8th World Water Forum in Brasilia in 2018, due to population growth, economic development, and changes in consumption patterns, the global demand for water resources is increasing by 1% per year, and this speed will be greatly accelerated in the future. There are currently 3.6 billion people living in water-deficient areas (for at least one month in a year), and that number could grow to between 4.8 billion and 5.7 billion by 2050. Although it seems that there is more water for agricultural irrigation than for industrial production, it is undeniable that with the growth of developing countries and emerging economies, this situation will not last long, and industrial water use will increase gradually. Under this trend, there may not be enough water for industrial production in the future. In a word, water demand has a very wide public coverage and will keep sustained growth. Therefore, the situation of water shortage will become more and more serious. According to Liu and Yang [4], two-thirds of China’s 669 cities are short of water, and some are extremely short of water.

In addition, with the development of society, water quality has gradually become an important factor affecting water resources. According to the data released by the World Water Week in Stockholm, 26–31 August 2012, 60% of the world’s ecosystem services are deteriorating [5]. According to China’s 2015 State of the Environment Report, approximately 35.5% of the state-controlled river sections distributed in 10 large river basins across China contained water graded class IV, V, or worse, and is deemed unsafe for human consumption. Only 9.1% of groundwater monitoring sites distributed in 202 cities had good water quality, while 61.3% were deemed poor or worse [6]. In addition, the water quality problem is not only becoming more severe locally, but also worldwide. With the development and utilization of natural resources by human beings, the ecological environment has been damaged, and with the further degradation of soil and water resources, the capacity of water storage eventually decreases, which not only leads to a substantial decline in the amount of water storage, but it also threatens the water quality. For example, since industrial wastewater and domestic sewage are discharged into surface water without treatment, groundwater pollution has further increased. At least 35 million people in Bangladesh and 6 million in India are estimated to be at risk from arsenic contamination in groundwater [7]. Saraswat et al. [8] believe that due to the severe deterioration of water quality, the available water resources would become increasingly unsuitable for human consumption, and even pose danger to human consumption.

It is estimated that nearly 80% of the world’s people are at risk of water security or water-related biodiversity [9]. Water security is broadly defined as “the provision of acceptable quantities and quality of water for health, livelihoods, ecosystems and production, and the acceptable level of water-related risks associated with humans, the environment and the economy” [10]. Many scholars have interpreted the definition of water security. For example, Westing [11] expanded the concept of comprehensive security, pointing out that comprehensive security includes two interrelated aspects, namely one is political security, which includes military, economic, and human rights, and the other is environmental security, which includes the protection and rational use of environmental resources. Li et al. [12] believed that water security is a state in which the natural hydrological cycle and human water-related activities would not cause water shortages, water pollution, flood, drought or other problems, and hinder the sustainable development of social economy and ecological environment. Shao et al. [13] divided water security into three aspects: flood management security, resource security and ecological security. Bakker [14] thought that water security promotes an acceptable level of water-related human and ecosystem risks, coupled with a sufficient quantity and quality of water to support livelihoods, national security, human health, and ecosystem services. Shao et al. [15] divided water security into economic development, flood control security, water supply security, and water environment security. Li et al. [12] divided water security into five aspects: water cycle, water environment, water ecology, water society, and water economy. Although all the studies above have promoted research on water security, there are few studies on the practical, comprehensive evaluation of regional water resources security, and the guiding role in understanding and solving water security problems at the national level is limited [16]. Water resource security actually involves social, economic, ecological, and other aspects. Its essence is not only that water supply capacity can meet reasonable water resources requirements, but also the different requirements of water quality standards; therefore, the scope of security evaluation should include water quantity and water quality security aspects.

At the same time, the evaluation methods of water security are also constantly applied and developed. For example, Ou et al. [17] combined entropy theory with fuzzy matter-element and established a fuzzy matter-element model based on entropy weight to evaluate drinking water security. Chang et al. [18] studied the impact of water resources security on urban development through the system dynamics model. Veettil and Mishra [19] used the concepts of blue and green water footprints, and they took human and climate factors into account to make a quantitative assessment of water security. Li et al. [20] put forward an improved fuzzy comprehensive evaluation method to evaluate the water environment safety of the three northeast provinces of China. Yin et al. [21] established a water resources security evaluation model based on the fuzzy mathematics theory to carry out the dynamic evaluation.

In addition, there is another class of methods available, i.e., decision making, which can be defined as identifying and selecting alternatives from a set of alternatives based on the decision makers’ (DMs) preferences. Xu et al. [22] used a novel multi-criteria decision making (MCDM) method to evaluate the water security. In the evaluation process, multiple criteria about water security are considered to rank the alternatives in different cities. Technique for Order Preference by Similarity to Ideal Solution (TOPSIS) is currently one of the most popular MCDM methods and works satisfactorily in various application areas [23,24,25,26]. TOPSIS was first developed by Hwang and Yoon [27] to solve MCDM problems with the basic principle to choose the alternative which has the shortest geometric distance from the positive ideal solution (PIS) and the farthest distance from negative ideal solution (NIS); however, it has an obvious disadvantage in dealing with practical problems: direct calculation of the collected data cannot accurately and reasonably reflect the actual situation. There are differences in the importance of each indicator, while TOPSIS still assumes that the weights of indicators are known and are generally equal when the importance of the indicators is different. Therefore, the Best–Worst Method (BWM), proposed by Rezaei [28,29], is employed and combined with TOPSIS to overcome this problem, for it derives the weights based on a pairwise comparison of the best and the worst criteria with the other criteria [30]. What is more, many of mankind’s choices and judgments are not in themselves perfectly rational, and human judgments including preferences are often vague. It is more appropriate to use fuzzy linguistic sets to reflect the preferences of experts in the evaluation process [31,32,33,34]. Therefore, triangular fuzzy linguistic sets are introduced in BWM here to more accurately describe the DMs’ qualitative judgments in reality.

To sum up, this paper focuses on combining fuzzy BWM and TOPSIS methods to form a hybrid fuzzy BWM-TOPSIS method for the evaluation of regional water resources security. In the proposed method, the BWM method considering triangular fuzzy linguistic sets is used to get the indicators’ weights in the evaluation system, based on which the TOPSIS method is employed to rank the water resources security measures of the regions (i.e., alternatives).

The general framework of regional water security evaluation is as follows: firstly, the key problem statement is introduced in Section 2. Section 3 proposes the hybrid fuzzy BWM-TOPSIS method and figures out how to combine these two approaches (i.e., fuzzy BWM and TOPSIS methods). In Section 4, the proposed method is applied in the water resources security evaluation of six regions in North China. A comparison analysis with the equal-weight TOPSIS method as well as the fuzzy BWM-AHP method and a sensitivity analysis for indicator weights are also carried out to verify the effectiveness of the proposed method. Additionally, some suggestions are given to improve the level of water resources security in this area. Finally, Section 5 gives the conclusion of this paper and remarks the future research direction.

## 2. Key Problem Statement

### 2.1. Comprehensive Consideration of Water Quantity and Water Quality

Water quantity security means meeting the reasonable human demand for water resources. Water quality security implies meeting the reasonable requirements of human beings and the ecological environment.

**Figure 1:**
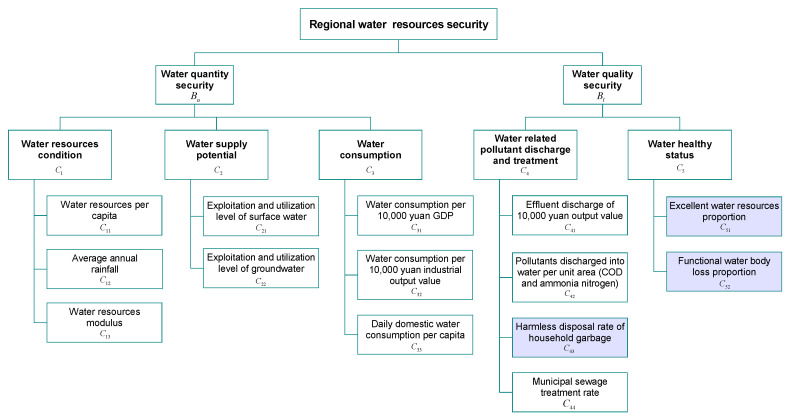
Regional water security evaluation indicator system.

If regional water resources security only considers the amount of water, it will pose a major threat to the ecological and social environment where people live. This results in the loss of high-quality water resources that can be used, although there are sufficient water resources, and this cannot meet the requirements of human beings and the ecological environment for water quality. If we only consider the quality of water, people will have high-quality water resources that can be used, but reasonable demand for water quantity cannot be met, and people will still be in a state of water scarcity. Therefore, in the water security evaluation system, both water quantity security and water quality security should be taken into account, which is the main idea of this paper. In this paper, the indicators of water quantity security includes water resources per capita, average annual rainfall, water resources modulus, exploitation and utilization level of surface water, exploitation and utilization level of groundwater, water consumption per 10,000 yuan GDP, water consumption per 10,000 yuan industrial output value, and daily domestic water consumption per capita, which are the main key factors that reflect water quantity security [19,35]. The indicators of water quality security include effluent discharge of 10,000 yuan output value, pollutants discharged into water per unit area (i.e., COD and ammonia nitrogen), municipal sewage treatment rate, harmless disposal rate of household garbage, excellent water resources proportion, and functional water body loss proportion. Most of these indicators are considered in many studies [35,36,37] as the main factors that reflect water quality security; however, to the best of the authors’ knowledge, the last three are seldom taken into account. Based on the above description, a comprehensive evaluation indicator system of water security is established and shown in Figure 1.

**Figure 2:**
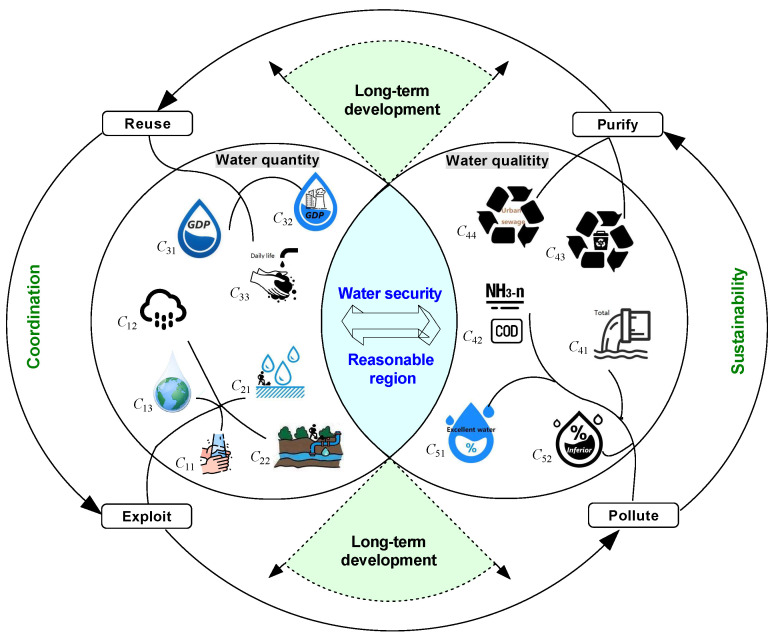
Regional water security evaluation system.

With the increase of the initial exploitation and utilization of surface and groundwater resources, the pollution level in water resources is deepened. Among them, effluent discharge of 10,000 yuan output value, the main pollutants discharged into water (i.e., COD and ammonia nitrogen), excellent water resources proportion, and functional water body loss proportion can reflect the polluted situation of water in a certain region. In order to solve this pollution situation and realize sustainable development, it is necessary to conduct harmless treatment of domestic garbage and sewage. At the same time, these purified water resources can be reused for daily life and industrial production so as to reduce the exploitation of all kinds of water resources. Therefore, it can coordinate the relationship between the reused water resources and the initial exploitation of water resources and better protect the water ecological environment. From this, we can see that water quality security and water quantity security are inseparable in water resources. Only when water quantity security and water quality security are both considered comprehensively can long-term, sustainable development of the water resources system be realized (see Figure 2).

### 2.2. Description of New Water Quality Indicators

As mentioned above, three indicators (i.e., harmless disposal rate of household garbage, excellent water resources proportion, functional water body loss proportion) are rarely considered in the previous studies. The reasons why they are included in the evaluation indicator system in this paper are listed below.

- Household garbage refers to the discarded leftovers, paper, plastic, glass, batteries, and fluorescent tubes in daily life. Some of the wastes contain toxic or harmful substances that are difficult to deal with, especially white pollution (i.e., waste food plastic bags) because of its difficult degradation. This pollution can be seen out in the open along roads or ditches in urban and rural areas, which will not only affect the surrounding environment but will also be washed into rivers or lakes during rainfall. It will seriously affect the safety of people’s living and production of water in downstream areas [38]. Therefore, harmless disposal of household garbage plays a positive role in protecting the surrounding water and groundwater resources, which should certainly be considered as an indicator for water quality.
- Excellent water resources proportion refers to the proportion of water bodies with a water quality grade of I-III (water quality grades are determined by China’s National Environmental Quality Standards for Surface Water), which generally refers to headwater, centralized domestic water, surface water source protection area, fishery water area, etc. It can directly reflect the quality of water, which is of great significance to judge the security of water quality and should be considered.
- Functional water body loss proportion refers to the proportion of water with water quality level lower than class V (generally refers to agricultural water or scenic spot water) [39]. The pollution degree of water in this proportion exceeds class V and is in an inferior state, thus losing the use function of water resources. Due to the intervention of some harmful substances, water bodies can change their chemical, physical, biological, or radioactive characteristics, which may endanger human health or destroy the ecological environment. This leads to water quality deterioration, thus affecting the effective use of water; therefore, it is taken into consideration as an essential indicator for water quality security.

## 3. Evaluation Methodology for Regional Water Resources Security

This section introduces the evaluation methodology including the general framework, the evaluation indicators, and the fuzzy BWM-TOPSIS method.

### 3.1. General Framework

The water resources security indicator system includes two primary subsystems, namely, water quantity security ( denoted as $B_{n}$) and water quality security ( denoted as $B_{l}$). For $B_{n}$, a few aspects are included, namely, water resources condition (denoted as $C_{1}$), water supply potential (denoted as $C_{2}$), and water consumption (denoted as $C_{3}$). For $B_{l}$, three aspects are involved, namely, water-related pollutant discharge and treatment (denoted as $C_{4}$) and water health status (denoted as $C_{5}$). The following principles should be followed in the selection of special indicators:

- The principle of integrity. A complete water security evaluation indicator system is put forward according to the regional characteristics with full consideration of all factors about water quantity security and water quality security.
- A combination of qualitative and quantitative aspects. The water security evaluation indicator system should try to select quantifiable indicators, and the important indicators that are difficult to quantify can adopt qualitative expression.
- The principle of comparability. In order to make the evaluation results comparable in different regions, the concepts and calculation method of indicators should be standardized.
- The principle of operability. The indicators about regional water resources security should be concise and clear in concept, and the data should be of good accessibility and avoid being too cumbersome, which can fully show the actual degree of regional water resources security and is conducive to its improvement.
- The principle of mutual exclusion. It should be ensured that there is no concept or content of coincidence among indicators in the evaluation system, and there is no intersection between water quantity and water quality subsystems.

### 3.2. Introduction of Evaluation Indicators

The security evaluation indicator system of regional water resources covers a wide range of contents. In order to better represent the security degrees of the designated regions, the evaluation system is divided into two primary subsystems with significant characteristics: water quantity security and water quality security.

#### 3.2.1. Water Quantity Security

- Water resources condition ($C_{1}$)

Water resources condition is about water resources per capita, average annual rainfall, and the water resources modulus in a region, which helps in creating a general understanding of the water resources status in the designated region.

Water resources per capita is a measure of the degree of water resources shortage in a certain region from the perspective of per capita, reflecting not only the overall water quantity but also the state of available water for each individual in the region. Assume that the total amount of water resources is represented by $T_{wr}$, and the total population is devoted by $T_{p}$, then water resources per capita (i.e., $C_{11}$) can be defined as

(1) $$\begin{array}{c} C_{11}=\frac{T_{wr}}{T_{p}}. \end{array}$$

Average annual rainfall is the sum of years of rainfall in a region divided by the number of years. The average annual rainfall can reflect the situation of local water resources. Let $C_{12}$ represent the average annual rainfall, $R_{s}$ represent the sum of years of rainfall in the region, and $N_{y}$ represent the number of years, then $C_{12}$ is defined as

(2) $$\begin{array}{c} C_{12}=\frac{R_{s}}{N_{y}}. \end{array}$$

Water resources modulus refers to the total amount of local water resources divided by the area of the region, which can directly reflect the average level of water resources in the region and avoid the large difference in total amount of water resources caused by the different evaluation regions. Let $C_{13}$ denote the modulus of water resources, suppose that the total amount of water resources is expressed by $T_{wr}$, and area of evaluation region is represented by $A_{e}$; therefore

(3) $$\begin{array}{c} C_{13}=\frac{T_{wr}}{A_{e}}. \end{array}$$

- Water supply potential ($C_{2}$)

Water supply potential is mainly used to describe the water supply capacity and the degree of water exploitation and utilization. It has a potential and deep-seated impact on water quantity security.

Exploitation and utilization level of surface water refers to the ratio of surface water supply to total surface water. It can indirectly reflect the amount of surface water exploitation and the future development prospects of surface water exploitation. The total regional surface water supply is expressed as $W_{ss}$, the total surface water is denoted as $W_{ts}$; therefore, the exploitation and utilization level of surface water $C_{21}$ can be calculated using the following equation:

(4) $$\begin{array}{c} C_{21}=\frac{W_{ss}}{W_{ts}}\times 100\%. \end{array}$$

Exploitation and utilization level of groundwater refers to the ratio of the water supply of groundwater to the total amount of groundwater. It can indirectly reflect the exploitation amount of groundwater and the development prospect of groundwater resources exploitation in the future. The groundwater supply is expressed as $W_{gs}$, the total amount of groundwater is denoted as $W_{tg}$; therefore, the exploitation and utilization level of groundwater $C_{22}$ can be given as follows:

(5) $$\begin{array}{c} C_{22}=\frac{W_{gs}}{W_{tg}}\times 100\%. \end{array}$$

- Water consumption ($C_{3}$)

In the process of daily production and life, water consumption, to a large extent, can intuitively describe the regional economic level and water consumption living standards. Water consumption is inseparable from the two aspects of economy and life.

Water itself is closely related to economic development. Among them, water consumption per 10,000 yuan GDP (i.e., $C_{31}$) and water consumption per 10,000 yuan industrial output value (i.e., $C_{32}$) can indirectly reflect the relationship between economic level and water consumption level in a region. Total water consumption is expressed by $W_{tc}$, 10,000 yuan of GDP is denoted by GDP, total industrial water consumption is written as $W_{ic}$, and 10,000 yuan industrial output value is represented as $V_{io}$. Therefore, $C_{31}$ and $C_{32}$ can be obtained by the following formulas:

(6) $$\begin{array}{c} C_{31}=\frac{W_{tc}}{GDP}, \end{array}$$

(7) $$\begin{array}{c} C_{32}=\frac{W_{ic}}{V_{io}}. \end{array}$$

Daily domestic water consumption per capita reflects the security of domestic water. The total domestic water consumption is represented as $D_{t}$, and the total population is denoted as $T_{p}$. The number of days is recorded as 365 for one year, so the daily domestic water consumption per capita (expressed as $C_{33}$) can be defined as

(8) $$\begin{array}{c} C_{33}=\frac{D_{t}}{T_{P}\times 365}. \end{array}$$

#### 3.2.2. Water Quality Security

To a great extent, water quality security is affected by sewage treatment and disposal as well as river water pollution. Better water quality conditions are conducive to the improvement of the whole ecological environment, and they also play an important role in maintaining water security. By reducing the discharge of substandard sewage and strengthening the protection of the river from pollution higher than its self recovery capacity, water quality security can be effectively guaranteed and will develop in a beneficial direction.

- Water-related pollutant discharge and treatment ($C_{4}$)

The major factors affecting water quality security in cities mainly include the effluent discharge per 10,000 yuan GDP, pollutants discharged into the water body per unit area, the municipal domestic sewage treatment rate, and the municipal domestic garbage disposal rate. The status of water-related pollutant discharge and treatment (denoted as $C_{4}$) is also mainly considered from these four aspects.

Water quality and health status can be reflected by the effluent discharge per 10,000 yuan GDP (i.e., $C_{41}$). Let $E_{td}$ denote the total effluent discharge, GDP represent 10,000 yuan GDP, then $C_{41}$ can be expressed as follows:

(9) $$\begin{array}{c} C_{41}=\frac{E_{td}}{GDP}. \end{array}$$

Another important indicator to measure water quality degree is the amount of pollutants discharged into the water per unit area. Pollutants discharged into the water mainly include chemical oxygen demand (COD) and ammonia nitrogen. COD is an important indicator of organic pollution of a water body, while ammonia nitrogen reflects nonorganic pollutants of a water body. The less COD and ammonia nitrogen, the healthier the water. Let $P_{t}$ be the amount of pollutants (COD and ammonia nitrogen) discharged into the water, $A_{e}$ be area of evaluation; pollutants discharged into water per unit area (denoted as $C_{42}$) can be calculated as follows:

(10) $$\begin{array}{c} C_{42}=\frac{P_{t}}{A_{e}}. \end{array}$$

As mentioned above, the treatment rate of harmless municipal solid waste should be considered in the water quality subsystem. Assume that the total discharge amount of urban household garbage is expressed as $G_{t}$, while the garbage that has reached the standard of emission is denoted as $G_{a}$. The treatment rate of harmless municipal solid waste (denoted as $C_{43}$) can be defined as follows:

(11) $$\begin{array}{c} C_{43}=\frac{G_{a}}{G_{t}}\times 100\%. \end{array}$$

The wastewater produced by human life is an indispensable part of wastewater treatment and an important part in measuring the security of water quality. Therefore, the municipal sewage treatment rate (denoted as $C_{44}$) should be considered in the water quality security subsystem. Suppose that the total sewage discharge is expressed by $S_{d}$, and the amount of urban sewage that enters the sewage treatment plant for treatment is represented by $S_{t}$; therefore

(12) $$\begin{array}{c} C_{44}=\frac{S_{t}}{S_{d}}\times 100\%. \end{array}$$

- Water health status ($C_{5}$)

The water health level of a river can directly judge the water quality security degree of a river basin. The following two kinds of water bodies in the water quality monitoring section of the river can intuitively present the water health level.

Excellent water resources proportion (i.e., $C_{51}$) refers to the proportion of a water body whose quality is up to or better than class III in the total water body. Let $W_{g}$ represent the amount of excellent water resources in a region, $W_{t}$ denotes the total water body of the same region; therefore

(13) $$\begin{array}{c} C_{51}=\frac{W_{g}}{W_{t}}. \end{array}$$

Functional water body loss proportion (i.e., $C_{52}$) refers to the proportion of a water body with inferior class V water quality in the total water body of the region. Let $W_{b}$ denote the water body with inferior class V water quality, $W_{t}$ represents the total water body of the region; therefore

(14) $$\begin{array}{c} C_{52}=\frac{W_{b}}{W_{t}}. \end{array}$$

### 3.3. Fuzzy BWM-TOPSIS Method

**Figure 3:**
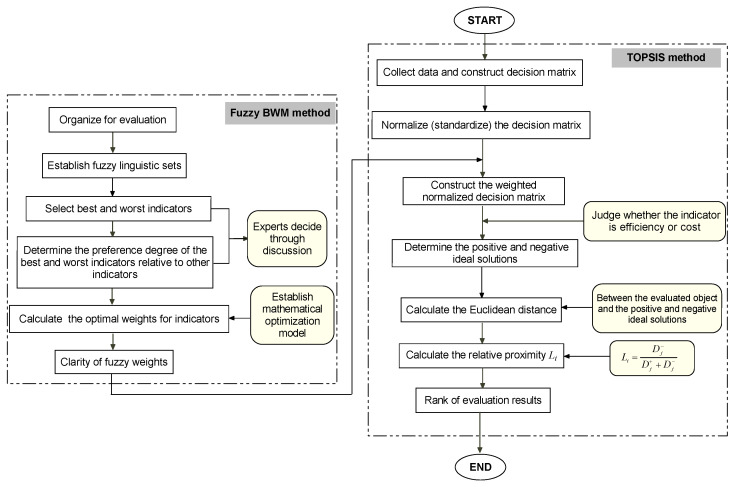
Fuzzy BWM-TOPSIS method framework.

In this section, a fuzzy BWM-TOPSIS method will be proposed for evaluating regional water resources security, and its framework can be seen in Figure 3.

#### 3.3.1. Fuzzy Linguistic Terms for Describing Referential Importance Degree

The evaluator is an expert with rich experience in water resources coordination; they makes linguistic judgments on the importance of water resources security indicators. Since triangular fuzzy linguistic terms can more accurately express human beings’ ideas than traditional single linguistic terms [31], they are used in this paper to represent the experts’ assessments on different water resources security indicators.

In this paper, according to the following BWM method, the evaluator will select a best indicator and a worst indicator and then compare the importance of other indicators with them using triangular fuzzy linguistic terms shown in Table 1. In this paper, five fuzzy linguistic terms are used by the expert to describe the referential importance degree, which include equally important (EI), weakly important (WI), fairly important (FI), very important (VI), and absolutely important (AI). For example, suppose that the best indicator chosen by the evaluator is $C_{52}$, and it is used to compare with indicator $C_{11}$, the expert may give an expression “The degree of importance of the best indicator $C_{52}$ compared to $C_{11}$ is weakly important”. Then, a triangular fuzzy number (2/3,1,3/2) can be used to describe the referential importance degree.

#### 3.3.2. Fuzzy BWM for Weight Determination

The BWM method obtains weights based on pairwise comparisons of the best and worst indicators with other indicators. Because BWM needs less comparative data and can be combined with other multi-criteria decision-making methods, it can be used in more and more practical situations [29]. In this paper, triangular fuzzy linguistic terms are used to describe the referential importance degrees as described in the above section. The graded mean integration representation (denoted as $GM(\tilde{\xi })$) of a triangular fuzzy number (denoted as $\tilde{\xi }$) [31] is used to determine the ranking of the triangular fuzzy number, which is calculated using the following formula:

(15) $$\begin{array}{c} GM\left(\tilde{\xi }\right)=\frac{l+4\times c+u}{6}, \end{array}$$

where $\tilde{\xi }=\left(l,c,u\right)$, *l*, *c*, *u* respectively represent the lower bound, most possible value, and upper bound of the judged object.

For the sake of convenience, suppose there are *m* indicators (denoted as $V_{j}\;\left(j=1,2,\cdots ,m\right)$). Based on the above description, the steps of fuzzy BWM are listed as follows:

**Step 1.** Select the best indicator $V_{B}$ and the worst indicator $V_{W}$ in the indicator set $\left\{V_{1},V_{2},\cdots ,V_{m}\right\}$.

**Step 2.** Execute the fuzzy reference comparisons for the best indicator. Table 1 is used to determine the fuzzy preference degree of the optimal indicator compared with other indicators, and a comparison vector is constructed:

(16) $$\begin{array}{c} {\tilde{A}}_{B}=\left\{{\tilde{a}}_{B1},{\tilde{a}}_{B2},\cdots ,{\tilde{a}}_{Bm}\right\}, \end{array}$$

where ${\tilde{a}}_{Bj}=\left(l_{Bj},c_{Bj},u_{Bj}\right)$ is represented by a fuzzy number, and it represents the fuzzy preference of the best indicator $V_{B}$ over *j*th indicator; it is clear that ${\tilde{a}}_{BB}=1$.

**Step 3.** Execute the fuzzy reference comparisons for the worst indicator. Determine the preference degree of all other indicators compared with the worst indicator and construct a comparison vector:

(17) $$\begin{array}{c} {\tilde{A}}_{W}=\left\{{\tilde{a}}_{1W},{\tilde{a}}_{2W},\cdots ,{\tilde{a}}_{mW}\right\}, \end{array}$$

where ${\tilde{a}}_{jW}=\left(l_{jW},c_{jW},u_{jW}\right)$ indicates the preference of the *j*th indicator over the worst indicator $V_{W}$; it is clear that ${\tilde{a}}_{WW}=1$.

**Step 4.** Find the optimal weights $\left({\tilde{\omega }}_{1}^{*},{\tilde{\omega }}_{2}^{*},\cdots ,{\tilde{\omega }}_{m}^{*}\right)$.

The optimal weight for an indicator is the one where, for each pair of ${\tilde{\omega }}_{B}/{\tilde{\omega }}_{j}$ and ${\tilde{\omega }}_{j}/{\tilde{\omega }}_{W}$, we have ${\tilde{\omega }}_{B}/{\tilde{\omega }}_{j}={\tilde{a}}_{Bj}$ and ${\tilde{\omega }}_{j}/{\tilde{\omega }}_{W}={\tilde{a}}_{jW}$. To satisfy these conditions for all *j*, we should find a solution where the maximum absolute differences of $|{\tilde{\omega }}_{B}/{\tilde{\omega }}_{j}-{\tilde{a}}_{Bj}|$ and $|{\tilde{\omega }}_{j}/{\tilde{\omega }}_{W}-{\tilde{a}}_{jW}|$ for all *j* are minimized. Considering the non-negativity and sum condition for the weights, the following problem results:

(18) $$\begin{array}{c} \begin{array}{c} \min\max_{f}\left\{\left|\frac{{\tilde{\omega }}_{B}}{{\tilde{\omega }}_{j}}-{\tilde{a}}_{Bj}\right|,\left|\frac{{\tilde{\omega }}_{j}}{{\tilde{\omega }}_{W}}-{\tilde{a}}_{jW}\right|\right\}\\ s.t.\left\{\begin{array}{c} \sum_{j=1}^{m}GM\left({\tilde{\omega }}_{j}\right)=1,\\ {\tilde{\omega }}_{j}\geq 0,\;\mathrm{for}\;\mathrm{all}\;j, \end{array}\right. \end{array} \end{array}$$

where ${\tilde{\omega }}_{B}=\left(l_{B}^{\omega },c_{B}^{\omega },u_{B}^{\omega }\right)$; ${\tilde{\omega }}_{j}=\left(l_{j}^{\omega },c_{j}^{\omega },u_{j}^{\omega }\right)$; ${\tilde{\omega }}_{W}=\left(l_{W}^{\omega },c_{W}^{\omega },u_{W}^{\omega }\right)$.

Suppose that $\tilde{\eta }=\left(l^{\eta },c^{\eta },u^{\eta }\right)$, considering $l^{\eta }<c^{\eta }<u^{\eta }$, let ${\tilde{\eta }}^{*}=\left(t^{*},t^{*},t^{*}\right)$, $t^{*}\leq l^{\eta }$, then model (18) is equivalent to the following model:

(19) $$\begin{array}{c} \begin{array}{c} \min\;{\tilde{\eta }}^{*}\\ s.t.\left\{\begin{array}{c} \left|\frac{\left(l_{B}^{\omega },c_{B}^{\omega },u_{B}^{\omega }\right)}{\left(l_{j}^{\omega },c_{j}^{\omega },u_{j}^{\omega }\right)}-\left(l_{Bj},c_{Bj},u_{Bj}\right)\right|\leq \left(t^{*},t^{*},t^{*}\right),\\ \left|\frac{\left(l_{j}^{\omega },c_{j}^{\omega },u_{j}^{\omega }\right)}{\left(l_{W}^{\omega },c_{W}^{\omega },u_{W}^{\omega }\right)}-\left(l_{jW},c_{jW},u_{jW}\right)\right|\leq \left(t^{*},t^{*},t^{*}\right),\\ \sum_{j=1}^{n}GM\left({\tilde{\omega }}_{j}\right)=1,\\ l_{j}^{\omega }\leq c_{j}^{\omega }\leq u_{j}^{\omega },\\ l_{j}^{\omega }\geq 0,\\ j=1,2,\cdots ,m. \end{array}\right. \end{array} \end{array}$$

By calculating the above model, the optimal fuzzy weight of indicator *j* (i.e., ${\tilde{\omega }}_{j}^{*}$) can be obtained.

#### 3.3.3. TOPSIS Method for Ranking the Alternatives

The basic idea of TOPSIS is to arrange the problems of comprehensive evaluation into a matrix, determine the ideal solution and negative ideal solution after normalization, and then calculate the distance between each evaluation alternative and the ideal solution or negative ideal solution. The basis of the ranking is that if the evaluation alternative is closest to the ideal solution, it is the best alternative. If the evaluation alternative is farthest from the ideal solution, it is the worst alternative [27].

Suppose that there are *n* regions (i.e., alternatives) (denoted as $R_{i}\;\left(i=1,2,\cdots ,n\right)$ ), the steps of the TOPSIS method in ranking the alternatives are listed as follows:

*Step 1.* Normalize (standardize) the indicator values. Suppose that $x_{i,j}$ represents the value of indicator *j* in region *i*, the normalized value of $x_{i,j}$ (denoted as $z_{i,j}$) can be calculated using the following formula:

(20) $$\begin{array}{c} z_{i,j}=\frac{x_{i,j}}{\sqrt{\sum_{i=1}^{n}x_{i,j}^{2}}}. \end{array}$$

*Step 2.* Use the fuzzy BWM method to calculate the weight (${\tilde{\omega }}_{j}^{*}|j=1,2,\cdots ,m$) of indicator *j*, and based on Equation (15), the determined value of the weight (determined as $GM({\tilde{\omega }}_{j}^{*})$) can be obtained.

*Step 3.* Construct the weighted normalized decision matrix. Let $y_{i,j}$ be the weighted normalized value of $x_{i,j}$, then $Y=(y_{i,j})$ can be represented as follows:

(21) $$Y=\left[\begin{array}{cccc} GM\left({\tilde{\omega }}_{1}^{*}\right)z_{1,1} & GM\left({\tilde{\omega }}_{2}^{*}\right)z_{1,2} & \cdots & GM\left({\tilde{\omega }}_{m}^{*}\right)z_{1,m}\\ GM\left({\tilde{\omega }}_{1}^{*}\right)z_{2,1} & GM\left({\tilde{\omega }}_{2}^{*}\right)z_{2,2} & \cdots & GM\left({\tilde{\omega }}_{m}^{*}\right)z_{2,m}\\ \vdots & \vdots & \ddots & \vdots \\ GM\left({\tilde{\omega }}_{1}^{*}\right)z_{n,1} & GM\left({\tilde{\omega }}_{2}^{*}\right)z_{n,2} & \cdots & GM\left({\tilde{\omega }}_{m}^{*}\right)z_{n,m} \end{array}\right].$$

*Step 4.* Judge whether the indicator belongs to benefit indicators set (denoted as $J_{1}$) or cost indicators set (denoted as $J_{2}$). For the positive ideal solution (denoted as $s_{i,j}^{+}$), the weighted normalized value of a benefit indicator should be maximized, while that of a cost indicator should be minimized. For the negative ideal solution (denoted as $s_{i,j}^{-}$), the weighted normalized value of a benefit indicator takes the minimum value, while that of a cost indicator takes the maximum value. Let $y_{i,j}^{\max}$ and $y_{i,j}^{\min}$ represent maximal and minimal weighted normalized values of indicator *j* in region *i*, respectively. Thus, the positive and negative ideal solutions can be obtained using Equations (22) and (23).

(22) $$s_{i,j}^{+}=\left\{\begin{array}{c} y_{i,j}^{\max},j\in J_{1},\\ y_{i,j}^{\min},j\in J_{2}. \end{array}\right.$$

(23) $$s_{i,j}^{-}=\left\{\begin{array}{c} y_{i,j}^{\min},j\in J_{1},\\ y_{i,j}^{\max},j\in J_{2}. \end{array}\right.$$

*Step 5.* Calculate the Euclidean distance between the evaluated region (i.e., alternative) *i* and the positive ideal solution (denoted as $D_{i}^{+}$), and that between the evaluated region (i.e., alternative) *i* and the negative ideal solution (denoted as $D_{i}^{-}$), according to the following expressions:

(24) $$\begin{array}{c} D_{i}^{+}=\sqrt{\sum_{j=1}^{m}{\left(s_{i,j}^{+}-y_{i,j}\right)}^{2}}, \end{array}$$

(25) $$\begin{array}{c} D_{i}^{-}=\sqrt{\sum_{j=1}^{m}{\left(s_{i,j}^{-}-y_{i,j}\right)}^{2}}. \end{array}$$

*Step 6.* Obtain the relative proximity $L_{i}$ between each region (i.e., alternative) *i* and the ideal solution using the following formula:

(26) $$\begin{array}{c} L_{i}=\frac{D_{j}^{-}}{D_{j}^{+}+D_{j}^{-}}. \end{array}$$

*Step 7.* Compare and obtain the ranking. $L_{i}$ is evaluated between 0 and 1; the closer $L_{i}$ is to 1, the closer it is to the optimal level of evaluation.

## 4. Case Study

**Figure 4:**
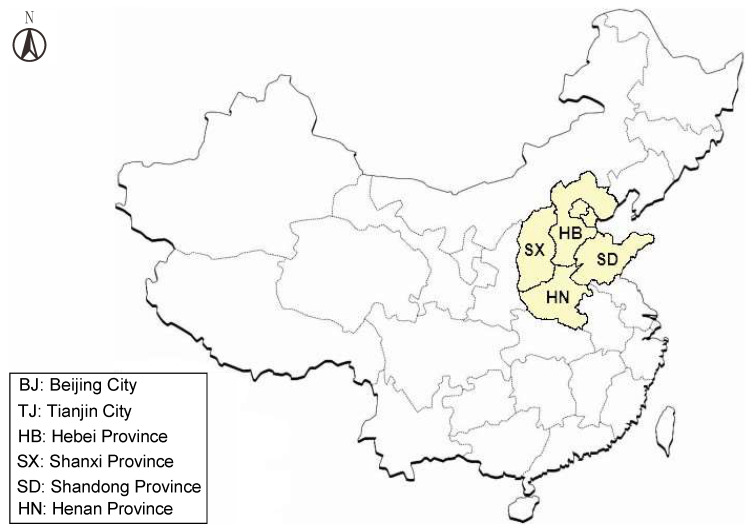
Location of evaluation regions.

The water resources security evaluation method based on hybid fuzzy BWM-TOPSIS is applied here to North China, with six provinces and cities as evaluation regions: Beijing City (denoted as BJ, i=1), Tianjin City (denoted as TJ, i=2), Hebei Province (denoted as HB, i=3), Shanxi Province (denoted as SX, i=4), Shandong Province (denoted as SD, i=5), and Henan Province (denoted as HN, i=6). The evaluation regions are shown in Figure 4, and the background and results of the evaluation are given below.

### 4.1. Case Description

China is a country with serious drought and water resource shortages. China’s total freshwater resources are 2.8 trillion m${}^{3}$, accounting for 6% of the world’s water resources. However, China’s per capita water resources are only 2300 m${}^{3}$, only 1/4 of the world average. In addition, China’s continental monsoon climate is so important that the water resources between the south and north are seriously unbalanced, resulting in the uneven distribution of water resources. At present, 47% of China’s total population is distributed in the northern region, which accounts for 45% of China’s GDP. In addition, the agricultural land in the southern region accounts for 35% of China’s land area, but its available water resources account for 81% of China’s total [40]. In particular, the six provinces and cities in North China considered in this case have a total area of about 700,000 km${}^{2}$ and a total population of about 346 million, accounting for about 25% of China’s population. Its total water resources are less than 4% of China’s total water resources; moreover, its per capita water resources are less than 13% of China’s average level, even lower than 5% of the world’s average level.

The security of water resources not only needs to consider the quantity, but it also should take the security of water quality in daily life into account. The economic development of the inland areas of North China mainly rely on traditional energy, heavy chemical industry, and traditional agriculture. For example, the coal industry of Shanxi Province used to be an important economic source of the whole province. Although the country has strengthened its environmental protection, a large amount of coal mining has caused serious damage to the water quality. Henan is a major agricultural province, with the main agricultural product output, ranking first in the country, and a cultivated land area of 6.87 million hectares, ranking second in the country. It has very large water resources consumption on agricultural land. Similarly, Hebei Province takes the resource processing type and the combination of industry and agriculture as the main economic structures. Moreover, Shandong Province and Tianjin City, as heavy chemical bases, occupy an important position in the national chemical industry. Once a chemical leak occurs, it will cause irreversible damage to nearby water sources and the offshore environment. Beijing, though dominated by service industry and high-tech industry, is the largest city in China, and water security is an important factor that cannot be ignored in this region. Once a security incident occurs, it will cause incalculable impacts. The industrial and agricultural activities to promote economic development and the activities of the service industry in these regions are heavily dependent on the water resources of the region. Compared with the coastal advantages of Shandong Province and Tianjin City, the situation of water shortages in other inland regions of North China is more severe. However, the highly developed industrial structure in coastal areas will also increase their dependence on seawater, and their impacts on water pollution may be greater than those in inland regions.

### 4.2. Data Collection

Most evaluation data in 2017 were obtained from the China National Bureau of statistics, which include total water resources, per capita water resources, surface water resources, underground water resources, total surface water supply, total underground water supply, area of provinces and cities, GDP in 2017, total industrial water consumption, modular industrial output value of each province, daily domestic water consumption per capita, harmless treatment rate of domestic waste, total wastewater discharge, and pollutants discharged into the water (COD and ammonia nitrogen). The information on the annual average rainfall in 2017 was acquired from the statistical yearbook of each region. The urban domestic sewage treatment rate came from the data of 2017 shown in the 2018 China Environmental Statistical Yearbook. The water resources proportion and functional water body loss proportion were obtained by consulting the 2017 Ecological Environment Bulletin of each region. Through data collection, the values of the indicators in six regions (i.e., alternatives) are shown in Table 2.

### 4.3. Interpretation of Results

Assume that $C_{42}$ and $C_{31}$ are selected as the best and the worst indicators, respectively, among the 14 indicators, and compare them with other indicators using the corresponding triangular fuzzy language judgment in Table 1. Table 3 shows the fuzzy ratings of the best indicator compared with each indicator. Table 4 shows the fuzzy ratings of all indicators compared with the worst indicator. The fuzzy weight of each indicator is calculated using model (19) as shown in Table 5. Then, the corresponding clear weights are calculated using Equation (15), which are shown in Table 6.

After collecting the data of each indicator, the decision matrix of Equation (21) is listed after standardization, and the weight of each indicator calculated above is used for weighting processing. Then, the corresponding positive and negative ideal solutions are selected after judging whether the indicator is a cost or benefit type. Then, Equations (24) and (25) are employed to calculate the Euclidean distance from each evaluation region (i.e., alternative) to the positive and negative ideal solution, and Equation (26) is utilized to calculate the relative proximity between each evaluation region (i.e., alternative) and the optimal scheme. The ranking result of the relative proximity $L_{i}\;\left(i=1,2,\cdots ,6\right)$ is as follows: $L_{2}\leq L_{1}\leq L_{3}\leq L_{5}\leq L_{4}\leq L_{6}$ (see Table 7). It can be seen from the ranking result of the relative proximity $L_{i}$ that, using the hybrid fuzzy BWM-TOPSIS method in Table 7, the overall water security level of Henan Province was the highest among the six regions (i.e., alternatives), Shanxi Province ranked second, followed by Shandong Province and Hebei Province, while Beijing City and Tianjin City ranked relatively low. Beijing City ranked second to last, and Tianjin City came last, its water security level was lowest.

### 4.4. Comparison Analysis

Previous studies lacked consideration of using a triangle fuzzy linguistic set to express the importance comparisons between water resource security indicators; they preferred the importance of indicators with equal weight. However, it is worth mentioning that different indicators should have different degrees of importance in the evaluation system, for some indicators have more significant impacts on water resources security. Therefore, the triangle fuzzy linguistic set is integrated in BWM here for generating different indicator weights for the TOPSIS method. A comparison of results of the proposed fuzzy BWM-TOPSIS method and the traditional equal-weight TOPSIS is shown in Table 7. It can be seen from Table 7 that, compared with the traditional TOPSIS method with equal indicator weights, $L_{2}$ ranked last, while $L_{6}$ ranked better than other regions in both methods. This shows that Henan Province had a steadily better performance in water resources security, while Tianjin City should pay more attention to the improvement of water resources security. For analyzing the differences of the two methods, the relative proximity values in all regions were different, and the rankings of Shandong Province and Shanxi Province changed in these two methods because the introduction of fuzzy linguistics assigned different weights of importance to the indicators. Compared with the equal-weight TOPSIS method, the water resources security ranking of Shanxi Province using the proposed hybrid fuzzy BWM-TOPSIS method rose, which was mainly because the weights of some indicators (e.g., pollutants discharged into water per unit area (COD and ammonia nitrogen), $C_{42}$; exploitation and utilization level of surface water, $C_{21}$) increased when they were assigned a higher degree of importance in the proposed hybrid fuzzy BWM-TOPSIS method due to their more significant impacts on water resources security. While the weights of some indicators (e.g., water consumption per 10,000 yuan GDP, $C_{31}$; water consumption per 10,000 yuan industrial output value, $C_{32}$) for which Shanxi Province had relatively worse performance decreased.

The Analytical Hierarchy Process (AHP) is one of the most widely adopted MCDM techniques as TOPSIS [41]; therefore, a comparison analysis between the proposed method with AHP method was conducted. For ensuring the fairness of comparison, the AHP method was given the same indicator weights obtained by fuzzy BWM method to avoid large differences in ranking caused by the different indicator weights, which means the proposed fuzzy BWM-TOPSIS method was compared with the fuzzy BWM-AHP method. The results are shown in Table 7. It can be seen from Table 7 that the rankings of Hebei Province and Beijing city changed, while the rankings of other regions remained unchanged for these two methods. The main reason is that TOPSIS and AHP have the following characteristics: for TOPSIS, the minimum distance to the positive ideal solution and the maximum distance to the negative ideal solution should be realized, and the ranking should be carried out according to the relative proximity degree, while AHP aims to determine the weights in regions (i.e., alternatives) hierarchy after obtaining the weights of indicators hierarchy and calculate the comprehensive index with the combination weight for sorting. What is more, the AHP method needs to decompose the decision-making problem into several subsystems, which requires a lot of pairwise comparisons, while the calculation process of the TOPSIS method is more convenient.

### 4.5. Sensitivity Analysis

**Figure 5:**
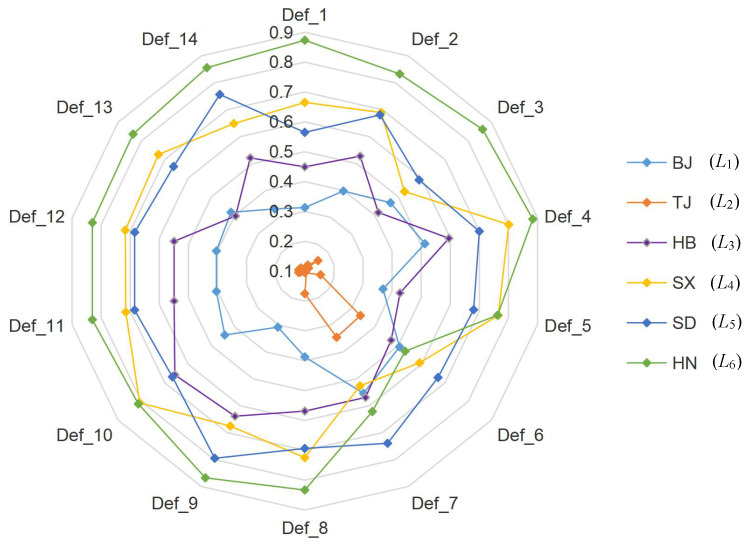
Sensitivity analysis results.

The sensitivity analysis of the final ranking of the scheme to the weights assigned to different indicators is conducted here so as to verify the effectiveness of the indicators’ weights calculated by the fuzzy BWM method. Fourteen groups of experiments were carried out. For the first experiment, based on the original indicator weights, the weight of indicator $C_{11}$ increased by 13%, while the sum of the other indicator weights reduced by 13%. Each of the other indicator weights will share this reduction percentage equally; in other words, all other indicator weights will be reduced by 1%. The same idea is applied to the other 13 groups of experiments on the other 13 indicators. Based on this consideration, the values of the final relative proximity degrees $L_{i}\;\left(i=1,2,\cdots ,6\right)$ in each experiment are calculated by Equations (21)–(26), and the rankings of the regions (i.e., alternatives) of 14 groups of experiments are operated in turn. The operation results are shown in Table 8 and Figure 5.

It can be seen from Figure 5 that the rankings of $L_{i}$ values of six regions (i.e., alternatives) were relatively stable. Although there were some changes in various provinces and cities, the results generated from a number of experiments showed that the relative proximity $L_{i}$ values of various provinces and cities fluctuated up and down only slightly. Therefore, the obtained results can be considered robust, to some extent. As shown in Figure 5, the relative proximity value of Beijing City (i.e., $L_{1}$) and that of Tianjin City (i.e., $L_{2}$) had lower rankings, especially Tianjin City which was maintained in the center of spider diagram, meaning it always came last in all the experiments. The relative proximity values of Hennan Province (i.e., $L_{6}$) and that of Shanxi Province (i.e., $L_{4}$) were in relatively higher places. It can also be noticed from Table 8 that the $L_{1}$ value of Beijing City ranked fifth in 11 instances, and the $L_{2}$ value of Tianjin City always ranked last. However, the relative proximity value of Henan Province (i.e., $L_{6}$) ranked first in 11 instances and second for 2. The value of Shanxi Province (i.e., $L_{4}$) ranked first once, second 9 times, and third 3 times. It should be noted that Shanxi Province has a large gap in the seventh sensitivity experiment, with its $L_{4}$ value dropping from second to fifth. The reason is that the seventh experiment increased the weight of water consumption per 10,000 yuan industrial output value (i.e., $C_{32}$) by 13%, and in terms of the performance of this indicator, Shanxi Province was the worst among the six regions. Synthesizing the above results, the water resources security levels in Henan Province and Shanxi Province are comparatively higher, while those of Tianjin City and Beijing City are comparatively lower in different weight combinations.

### 4.6. Suggestions

According to the collected indicator data and the final ranking results, some key problems of water resources are highlighted in North China.

**Figure 6:**
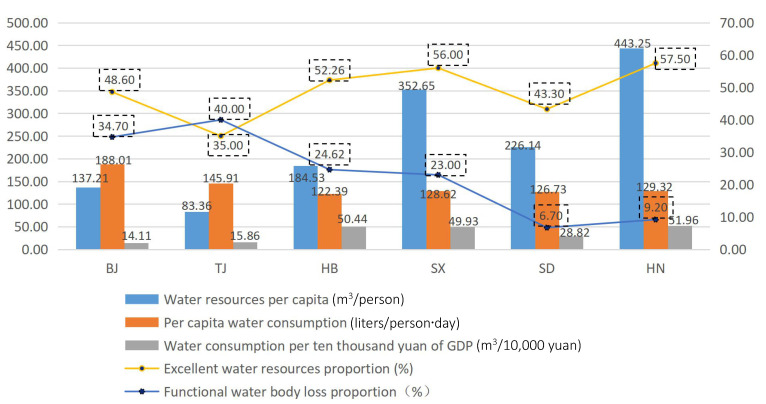
Situation of some indicators in North China.

First, from the perspective of water quantity security, the distribution of water resources among regions is uneven. Relatively speaking, the economic development of the region is highly dependent on the water resources of the region, the more developed a region is, the higher its population will be, and the less its corresponding per capita share will be, which will further aggravate regional water resource disparities. As shown in Figure 6, for example, Beijing City is relatively more developed than Henan Province, which can be seen from the water consumption per 10,000 yuan GDP. The value for Beijing City was as low as 14.11 m${}^{3}$/10,000 yuan, which is about 1/4 that of Henan Province; however, the the per capita water resources of Beijing City was 137.21 m${}^{3}$/person, which is about 1/3 that of Henan Pronvince. What is more, from Figure 6, Beijing has a very large population, its water resources per capita (137.21 m${}^{3}$/person) ranked last but once; however, its daily water consumption of residents (188.01 L/person·day) ranked first in all regions. This shows that the regional water resources security in Beijing City is in poor condition and also explains the reason that the water resources security ranking of Beijing City stayed lower all the time.

Second, from the aspect of water quality security, water resources are seriously polluted from a general view. As seen in Figure 6, water quality security situations in Tianjin and Beijing are worse than those in other regions, especially in Tianjin City. The proportion of excellent water resources was as low as 35.00%, which was the lowest among all regions; however, its functional water body loss proportion was as high as 40.00%, which was the worst among all regions. This shows that Tianjin has bad water quality security conditions, and some efficient measures should be taken to deal with these problems.

Integrating the above analysis, suggestions are put forward to promote the overall development of North China and make effective and reasonable use of water resources.

(1) Put forward the strategic plan of water use. The local government should optimize industrial structures, promote the adoption of innovative water-saving technology, and develop clean energy and other measures so as to improve water use and reduce water resources consumption and sewage discharge [42]. At the same time, it is suggested that investment in laboratory infrastructure should be increased to promote the assurance of water quality monitoring. The local government should also encourage stakeholders to comprehensively solve the causes of water damage and actively respond to the water protection measures formulated by the government [43,44,45]. Some policies or strategies should be adopted to coordinate the interrelated departments and stakeholders in order to achieve consensus among departments and individuals in reducing the harm human factors pose to the regional water resources environment.

(2) Crack down on factories and enterprises with severe water pollution, and slowly raise the standards for industrial waste water discharge. Water pollution is still an inevitable obstacle to the progress and development of modern society. Nearly 1/5 of water in rivers in North China cannot be used, and the unit area of pollutants discharged into the water is an important cause of water pollution; therefore, as China’s heavy industry base, the local governments in North China need to more strictly control industrial wastewater, and people should also play a role in supervising the local governments and enterprises.

(3) Focus on the development of seawater desalination technology to reduce the development and utilization of inland surface water and groundwater. With the increase in population and rapid development of the economy, the demand for water resources is constantly intensifying. In the process of urbanization, excessive exploitation of groundwater reduces the recycling of groundwater and intensifies damage to groundwater. Desalination technology could efficiently settle the shortage problem of water resources in North China (near the sea).

(4) Publicize the importance of water conservation, promote the installation of water-saving equipment in rural areas, and form good habits of recycling and water conservation, especially in the rural areas of villages and towns. At present, the awareness of water resources protection is still weak, and the water-saving facilities in a household are not complete. It should be noted that the potential for continuous development of water resources in the region is limited, while the potential for water conservation (i.e., multi-functional utilization of domestic water, industrial water reuse) is very large, so it is of significant importance to publicize water-saving ideas to the people. In addition, we can promote water-saving behaviors and mobilize people’s enthusiasm for water saving by appropriately raising water prices in different regions.

For the provinces and cities in North China, the following suggestions are put forward.

(1) Based on the evaluation results, Henan Province is closest to the optimal solution and farthest from the worst solution (i.e., ranking highest) among the six regions. Compared with the other five regions, Henan Province is rich in water resources, and its water quantity security and quality security are both at relatively better levels. However, its water consumption per 10,000 yuan GDP is highest among six regions (see Figure 6); therefore, there is still a lot of room to improve the efficiency of economic operation. As a big agricultural province, Henan can reduce the water consumption per 10,000 yuan GDP by increasing the water reuse rate in agriculture. Based on this, it can improve its water resources security level even further.

(2) The water resources security level in Shanxi Province ranks second, but as a large coal mining province, it should be noted that the garbage and sewage treatment rates in the province still need to be improved, for its two indicators (i.e., harmless disposal rate of household garbage, municipal sewage treatment rate) have relatively worse performances among the six regions. Some centralized sewage or garbage treatment plants can be built on the original grounds to improve the treatment rate of pollution so as to prevent the re-pollution of industrial wastewater. In addition, it can also be seen from the seventh experiment for sensitivity analysis in Table 8 and Figure 5 that the water consumption per 10,000 yuan industrial output value had a great influence on the ranking of Shanxi Province. The local government should increase publicity to enterprises to eliminate the behavior of wasting water in industry, and at the same time enterprises should also reduce their industrial water consumption by adopting more industrial water-saving facilities and enhancing the reuse of industrial water. Through the above measures, its weak points can be improved which will apparently enhance its performance on water resources security.

(3) Shandong Province, ranking third among the six regions of North China, has a relatively poor performance in both functional water body loss proportion and the pollutants discharged into water per unit area (COD and ammonia nitrogen), which are the main reasons for it ranking lower than Shanxi Province. Therefore, it is imperative to strictly control the re-pollution of water bodies and the discharge amount of pollutants to strengthen its performance on water resources security.

(4) Hebei Province, oriented towards heavy industry and ranking relatively in the middle in the whole region, should try to change its industrial structure to light industry gradually. At present, the wastewater and residue produced by heavy industry is relatively high, as the effluent discharge per 10,000 yuan GDP of Hebei Province is larger than that of Shandong Province and Henan Province, so more strict examination and purification measures should be taken in order to control pollution and improve its water quality security level. In addition, exploitation and utilization levels of surface water and groundwater are relatively high, especially for groundwater, which should be controlled to avoid damage to the ecological environment caused by over exploitation and to narrow the gap with the top two regions.

(5) Beijing City, as a region with wide concerns, had the penultimate comprehensive score. Some indicators were close to the worst line in North China, such as daily domestic water consumption per capita, the effluent discharge per 10,000 yuan GDP, proportion of excellent water resources and functional water body loss proportion, and exploitation and utilization level of surface water. Therefore, the local government not only needs to strengthen the protection of rivers, which can promote to control the water pollution and protect water resources environment, but also control surface water exploitation in the meantime. Moreover, it should be noted that the daily domestic water consumption per capita in Beijing City is much higher than that in other regions, which does not match the per capita water resources in Beijing City. Therefore, it may be necessary to raise the water price to encourage people’s daily water saving. All in all, it can be seen from the above analysis that Beijing City did not perform well in water quantity and quality security subsystems. The local government should take active and forceful measures to build a better city image in water resources security.

(6) Tianjin City, always ranking last among the six regions, had the lowest per capita water resources and over developed surface water resources with high load (216% surface water exploitation and utilization degree). Therefore, the local government should limit local water resources exploitation. It can alleviate the shortage of water resources by purchasing water resources intensive products. While depending on the South-to-North Water Diversion Project for water supply, it should also rely on government propaganda to promote water-saving awareness among the people. In addition, the lower water resources security level in Tianjin City is not only reflected in the amount of water, water quality security is also under serious condition. Since 40% of the river water in Tianjin City has lost its function, it is necessary to formulate a comprehensive prevention and control plan for water pollution, adhere to the principle of comprehensive control, and cooperate with the economic growth mode transformation, so as to improve the efficiency of pollution control. With consideration of its very poor performance in water resources security, Tianjing City cannot change its situation at the bottom of the ranking without attaching great importance on the aforementioned water quantity and water quality measures.

## 5. Conclusions and Future Research

The main claim of this paper is that regional water resource security is closely related to people’s lives and health, so ensuring regional water resources security level can improve people’s life quality. Based on the above consideration, this paper proposes a novel methodology for regional water resources security evaluation. Firstly, this paper divides regional water resources security system into water quantity and quality security subsystems involving social, economic, ecological, and environmental factors, based on which a regional water resources security indicator system is established. It is worth mentioning that the three key factors of the water quality security subsystem (i.e., harmless disposal rate of household garbage, excellent water resources proportion, and functional water body loss proportion) are highlighted and analyzed. Secondly, a fuzzy BWM-TOPSIS method is proposed to rank the regional water resources security of the regions (i.e., alternatives). In this method, triangular fuzzy linguistic set, which can more accurately express evaluator judgements on the importance degrees of indicators, is introduced in the BWM method to generate a fuzzy BWM method, through which the indicator weights can be obtained. Based on the generated indicator weights, the TOPSIS method is utilized to rank the regions (i.e., alternatives) through the formula of Euclidian distance between each region and the optimal/worst solution. Finally, the regional water resources security evaluation indicator system and the integrated fuzzy BWM-TOPSIS method are applied to conduct a case study on six regions in North China. A comparison analysis with equal-weight TOPSIS method and a sensitivity analysis on indicator weights are conducted to verify the practicality and efficiency of the proposed method. Furthermore, some managerial suggestions on improving the water resources security levels in the whole region and different provinces and cities of North China are given.

The multi-criteria decision-making method proposed in this paper is not only suitable for regional water resources security evaluation, but it could also be applicable to other evaluation problems. The future research direction could focus on studying and employing more complicated fuzzy linguistic environments (i.e., hesitant fuzzy linguistic environment, type-2 fuzzy environment) in the evaluation methodology. In addition, we will keep track of the latest available data of the water resources security evaluation system to analyze variation trends and also further the research.

## Figures and Tables

**Table 1:** Transformation rules of linguistic variables of decision makers.

Linguistic Variables	Membership Function
Equally important (EI)	(1, 1, 1)
Weakly important (WI)	(2/3, 1, 3/2)
Fairly important (FI)	(3/2, 2, 5/2)
Very important (VI)	(5/2, 3, 7/2)
Absolutely important (AI)	(7/2, 4, 9/2)

**Table 2:** Values of the indicators in 6 regions.

Indicator	*j*	Type	BJ	TJ	HB	SX	SD	HN
$C_{11}$ (m${}^{3}$/person)	j=1	B-I	137.21	83.36	184.53	352.65	226.14	443.25
$C_{12}$ (mm)	j=2	B-I	615.50	566.80	527.40	572.60	694.50	790.00
$C_{13}$ (m${}^{3}$/m${}^{2}$)	j=3	B-I	0.18	0.11	0.07	0.08	0.14	0.25
$C_{21}$ (%)	j=4	C-I	103.00	216.00	99.00	42.00	87.00	36.00
$C_{22}$ (%)	j=5	C-I	81.00	84.00	99.70	30.00	53.00	56.00
$C_{31}$ (m${}^{3}$/10,000 yuan)	j=6	C-I	14.11	15.86	50.44	49.93	28.82	51.96
$C_{32}$ (m${}^{3}$/10,000 yuan)	j=7	C-I	1.72	3.24	3.91	7.63	2.02	6.33
$C_{33}$ (liters/person·day)	j=8	C-I	188.01	145.91	122.39	128.62	126.73	129.31
$C_{41}$ (ton/10,000 yuan)	j=9	C-I	17.85	21.99	7.05	8.88	1.25	3.00
$C_{42}$ (ton/square kilometers)	j=10	C-I	5.34	8.97	2.96	1.44	3.80	2.95
$C_{43}$ (%)	j=11	B-I	99.90	94.40	99.80	94.90	100.00	99.70
$C_{44}$ (%)	j=12	B-I	97.5 0	92.60	97.80	92.60	97.00	96.90
$C_{51}$ (%)	j=13	B-I	48.60	35.00	52.26	56.00	43.30	57.50
$C_{52}$ (%)	j=14	C-I	34.70	40.00	24.62	23.00	6.70	9.20
Note: benefit indicator is represented as B-I, cost indicator is represented as C-I.								

**Table 3:** Fuzzy rating of the best indicator compared with each indicator.

Indicator	$C_{11}$	$C_{12}$	$C_{13}$	$C_{21}$	$C_{22}$
Best indicator $C_{42}$	WI (2/3,1,3/2)	VI (5/2,3,7/2)	FI (3/2,2,5/2)	WI (2/3,1,3/2)	WI (2/3,1,3/2)
Indicator	$C_{31}$	$C_{32}$	$C_{33}$	$C_{41}$	$C_{42}$
Best indicator $C_{42}$	AI (3/2,2,5/2)	VI (5/2,3,7/2)	VI (5/2,3,7/2)	WI (2/3,1,3/2)	EI (1,1,1)
Indicator	$C_{43}$	$C_{44}$	$C_{51}$	$C_{52}$	
Best indicator $C_{42}$	WI (2/3,1,3/2)	FI (3/2,2,5/2)	FI (3/2,2,5/2)	FI (3/2,2,5/2)	

**Table 4:** Fuzzy ratings of all indicators compared with the worst indicator.

Indicator	Worst Indicator $C_{31}$	Indicator	Worst Indicator $C_{31}$	Indicator	Worst Indicator $C_{31}$
$C_{11}$	VI (5/2,3,7)	$C_{31}$	EI (1,1,1)	$C_{43}$	VI (5/2,3,7/2)
$C_{12}$	WI (2/3,1,3/2)	$C_{32}$	WI (2/3,1,3/2)	$C_{44}$	FI (3/2,2,5/2)
$C_{13}$	FI (3/2,2,5/2)	$C_{33}$	WI (2/3,1,3/2)	$C_{51}$	FI (3/2,2,5/2)
$C_{21}$	VI (5/2,3,7/2)	$C_{41}$	VI (5/2,3,7)	$C_{52}$	FI (3/2,2,5/2)
$C_{22}$	VI (5/2,3,7/2)	$C_{42}$	AI (3/2,2,5/2)		

**Table 5:** Fuzzy weight of each indicator.

Indicator	$C_{11}$	$C_{12}$	$C_{13}$
Fuzzy weight	${\tilde{\omega }}_{1}=\left(0.0687,0.1020,0.1035\right)$	${\tilde{\omega }}_{2}=\left(0.0301,0.0402,0.0454\right)$	${\tilde{\omega }}_{3}=\left(0.0409,0.0673,0.0763\right)$
Indicator	$C_{21}$	$C_{22}$	
Fuzzy weight	${\tilde{\omega }}_{4}=\left(0.0687,0.1020,0.1035\right)$	${\tilde{\omega }}_{5}=\left(0.0687,0.1020,0.1035\right)$	
Indicator	$C_{31}$	$C_{32}$	$C_{33}$
Fuzzy weight	${\tilde{\omega }}_{6}=\left(0.0273,0.0309,0.0312\right)$	${\tilde{\omega }}_{7}=\left(0.0301,0.0402,0.0402\right)$	${\tilde{\omega }}_{8}=\left(0.0301,0.0402,0.0402\right)$
Indicator	$C_{41}$	$C_{42}$	$C_{43}$
Fuzzy weight	${\tilde{\omega }}_{9}=\left(0.0687,0.1020,0.1035\right)$	${\tilde{\omega }}_{10}=\left(0.0999,0.1145,0.1145\right)$	${\tilde{\omega }}_{11}=\left(0.0687,0.1020,0.1035\right)$
Indicator	$C_{44}$	$C_{51}$	$C_{52}$
Fuzzy weight	${\tilde{\omega }}_{12}=\left(0.0409,0.0673,0.0763\right)$	${\tilde{\omega }}_{13}=\left(0.0409,0.0673,0.0763\right)$	${\tilde{\omega }}_{14}=\left(0.0409,0.0673,0.0763\right)$

**Table 6:** Weight of each indicator.

Indicator	$C_{11}$	$C_{12}$	$C_{13}$	$C_{21}$	$C_{22}$	$C_{31}$	$C_{32}$
Indicator’s weight	0.0967	0.0394	0.0644	0.0967	0.0967	0.0304	0.0385
Indicator	$C_{33}$	$C_{41}$	$C_{42}$	$C_{43}$	$C_{44}$	$C_{51}$	$C_{52}$
Indicator’s weight	0.0385	0.0967	0.1121	0.0967	0.0644	0.0644	0.0644

**Table 7:** Results and comparison analysis.

Evaluation Region	Fuzzy BWM-TOPSIS		Equal Weight TOPSIS		Fuzzy BWM-AHP	
	$L_{i}$	Rank by $L_{i}$	$L_{i}^{{}^{\prime }}$	Rank by $L_{i}^{{}^{\prime }}$	AHP Score	Rank
BJ (i=1)	0.4081	V	0.4717	V	0.1368	IV
TJ (i=2)	0.1400	VI	0.2800	VI	0.1079	VI
HB (i=3)	0.5437	IV	0.4945	IV	0.1351	V
SX (i=4)	0.7035	II	0.5740	III	0.2122	II
SD (i=5)	0.6852	III	0.6899	II	0.1924	III
HN (i=6)	0.8151	I	0.7003	I	0.2155	I

**Table 8:** Experiments for sensitivity analysis.

Expt. no. *k*	Definition (Def_*k*)	$L_{i}$ Value of Each Alternative						Ranking
		BJ ($L_{1}$)	TJ ($L_{2}$)	HB ($L_{3}$)	SX ($L_{4}$)	SD ($L_{5}$)	HN ($L_{6}$)	
1	Def_1	0.3130	0.0954	0.4493	0.6651	0.5648	0.8734	$L_{6}$>$L_{4}$>$L_{5}$>$L_{3}$>$L_{1}$>$L_{2}$
2	Def_2	0.3979	0.1228	0.5276	0.6899	0.6808	0.8330	$L_{6}$>$L_{4}$>$L_{5}$>$L_{3}$>$L_{1}$>$L_{2}$
3	Def_3	0.4675	0.1567	0.4153	0.5376	0.5900	0.8619	$L_{6}$>$L_{5}$>$L_{4}$>$L_{1}$>$L_{3}$>$L_{2}$
4	Def_4	0.5129	0.0852	0.5955	0.8012	0.6999	0.8837	$L_{6}$>$L_{4}$>$L_{5}$>$L_{3}$>$L_{1}$>$L_{2}$
5	Def_5	0.3688	0.1542	0.4274	0.7649	0.6804	0.7626	$L_{4}$>$L_{6}$>$L_{5}$>$L_{3}$>$L_{1}$>$L_{2}$
6	Def_6	0.5063	0.3385	0.4703	0.5922	0.6710	0.5310	$L_{5}$>$L_{4}$>$L_{6}$>$L_{1}$>$L_{3}$>$L_{2}$
7	Def_7	0.5542	0.3458	0.5692	0.5263	0.7397	0.6217	$L_{5}$>$L_{6}$>$L_{3}$>$L_{1}$>$L_{4}$>$L_{2}$
8	Def_8	0.3870	0.1754	0.5686	0.7244	0.6936	0.8329	$L_{6}$>$L_{4}$>$L_{5}$>$L_{3}$>$L_{1}$>$L_{2}$
9	Def_9	0.3080	0.0827	0.6390	0.6756	0.7954	0.8681	$L_{6}$>$L_{5}$>$L_{4}$>$L_{3}$>$L_{1}$>$L_{2}$
10	Def_10	0.4430	0.0837	0.6570	0.8076	0.6675	0.8132	$L_{6}$>$L_{4}$>$L_{5}$>$L_{3}$>$L_{1}$>$L_{2}$
11	Def_11	0.4039	0.1210	0.5492	0.7153	0.6849	0.8301	$L_{6}$>$L_{4}$>$L_{5}$>$L_{3}$>$L_{1}$>$L_{2}$
12	Def_12	0.4039	0.1210	0.5492	0.7170	0.6846	0.8301	$L_{6}$>$L_{4}$>$L_{5}$>$L_{3}$>$L_{1}$>$L_{2}$
13	Def_13	0.4163	0.1170	0.3966	0.7270	0.6623	0.8361	$L_{6}$>$L_{4}$>$L_{5}$>$L_{3}$>$L_{1}$>$L_{2}$
14	Def_14	0.3314	0.0972	0.5211	0.6484	0.7568	0.8563	$L_{6}$>$L_{5}$>$L_{4}$>$L_{3}$>$L_{1}$>$L_{2}$

Note: Def${}_{k}$—$GM({\tilde{\omega }}_{k}^{*})$+13%, $GM({\tilde{\omega }}_{j}^{*})$−1%, j,k=1,2,⋯,14, j≠k.
